# 7.0-T Magnetic Resonance Imaging Characterization of Acute Blood-Brain-Barrier Disruption Achieved with Intracranial Irreversible Electroporation

**DOI:** 10.1371/journal.pone.0050482

**Published:** 2012-11-30

**Authors:** Paulo A. Garcia, John H. Rossmeisl, John L. Robertson, John D. Olson, Annette J. Johnson, Thomas L. Ellis, Rafael V. Davalos

**Affiliations:** 1 Bioelectromechanical Systems Lab, School of Biomedical Engineering and Sciences, Virginia Tech-Wake Forest University, Blacksburg, Virginia, United States of America; 2 Neurology and Neurosurgery, Virginia-Maryland Regional College of Veterinary Medicine, Blacksburg, Virginia, United States of America; 3 Biomedical Sciences and Pathobiology, Virginia-Maryland Regional College of Veterinary Medicine, Blacksburg, Virginia, United States of America; 4 Center for Biomolecular Imaging, Wake Forest University School of Medicine, Winston-Salem, North Carolina, United States of America; 5 Department of Radiology, Wake Forest University School of Medicine, Winston-Salem, North Carolina, United States of America; 6 Department of Neurosurgery, Wake Forest University School of Medicine, Winston-Salem, North Carolina, United States of America; The Ohio State University Medical Center, United States of America

## Abstract

The blood-brain-barrier (BBB) presents a significant obstacle to the delivery of systemically administered chemotherapeutics for the treatment of brain cancer. Irreversible electroporation (IRE) is an emerging technology that uses pulsed electric fields for the non-thermal ablation of tumors. We hypothesized that there is a minimal electric field at which BBB disruption occurs surrounding an IRE-induced zone of ablation and that this transient response can be measured using gadolinium (Gd) uptake as a surrogate marker for BBB disruption. The study was performed in a Good Laboratory Practices (GLP) compliant facility and had Institutional Animal Care and Use Committee (IACUC) approval. IRE ablations were performed *in vivo* in normal rat brain (n = 21) with 1-mm electrodes (0.45 mm diameter) separated by an edge-to-edge distance of 4 mm. We used an ECM830 pulse generator to deliver ninety 50-μs pulse treatments (0, 200, 400, 600, 800, and 1000 V/cm) at 1 Hz. The effects of applied electric fields and timing of Gd administration (−5, +5, +15, and +30 min) was assessed by systematically characterizing IRE-induced regions of cell death and BBB disruption with 7.0-T magnetic resonance imaging (MRI) and histopathologic evaluations. Statistical analysis on the effect of applied electric field and Gd timing was conducted via Fit of Least Squares with α = 0.05 and linear regression analysis. The focal nature of IRE treatment was confirmed with 3D MRI reconstructions with linear correlations between volume of ablation and electric field. Our results also demonstrated that IRE is an ablation technique that kills brain tissue in a focal manner depicted by MRI (n = 16) and transiently disrupts the BBB adjacent to the ablated area in a voltage-dependent manner as seen with Evan's Blue (n = 5) and Gd administration.

## Introduction

In spite of aggressive therapy, the median survival for the majority of patients with glioblastoma multiforme (GBM) is approximately 15 months [1]. One of the reasons for poor survival is that tumor cells diffusely infiltrate the brain parenchyma [2]. Effective treatment of GBM may be limited by inefficient intracellular delivery of chemotherapy. Most agents demonstrating *in vitro* cytotoxic effects against glial tumors do not cross the blood-brain-barrier (BBB) *in vivo*. Although the BBB is compromised in portions of GBM, there is convincing evidence that these heterogeneous tumors frequently contain areas of infiltrative tumor which do not show enhancement, and therefore which are not likely affected by systemic chemotherapeutic agents [3], [4]. A technique that uniformly increases BBB permeability and therefore delivery of cytotoxic agents into tumors, may yield improved tumor control [5].

Electrochemotherapy (ECT) is a technique that uses pulsed electric fields to facilitate the uptake of chemotherapeutic agents, which then induces tumor cell death [6], [7], [8]. Therapeutic irreversible electroporation (IRE) is an emerging technology that also uses pulsed electric fields to produce non-thermal ablation of tumors [9], [10], [11], [12]. IRE creates a sharply delineated volume of ablated tissue, with sub-millimeter resolution [13]. IRE treatments involve inserting needle-like electrodes into the tumor and delivering a series of low-energy pulses to permanently destabilize the cell membranes, inducing death without thermal damage [9], [14]. IRE primarily affects the cell membrane of target cells, sparing important tissue components such as major blood vessels and extracellular matrix [15]. It has been demonstrated that IRE safely disrupts the BBB and precisely ablates normal and neoplastic brain tissue [16], [17], [18], [19].

For pulsed electric field therapies such as ECT and IRE to be therapeutically predictable in the brain a systematic study characterizing the duration and extent of BBB disruption and cell death achieved by different pulse parameters is needed. We hypothesized that there is a minimal electric field at which BBB disruption occurs surrounding an IRE-induced zone of ablation and that this transient response can be measured using Gd uptake as a surrogate marker for BBB disruption [5], [20], [21], [22]. This phenomenon, if present, may improve delivery of otherwise poorly diffusible anti-tumoral agents across the BBB into regions containing microscopic glioma infiltrates. Thus, irreversible electroporation enhanced pharmacotherapy may be a much more effective treatment for GBM due to its ability to destroy tumor cells within a discrete zone while increasing susceptibility to exogenous agents outside the zone of ablation. Using IRE to either destroy the tumor or increase the delivery of therapeutic agents to facilitate treatment of surrounding “at risk” tumor margins could therefore result in improved tumor control by treating the area in which most recurrences occur.

## Materials and Methods

The funders had no role in study design, data collection and analysis, decision to publish, or preparation of the manuscript.

### *In vivo* Irreversible Electroporation Protocol

The study was performed in a Good Laboratory Practices (GLP) compliant facility and was approved by the Institutional Animal Care and Use Committee (IACUC) at the Wake Forest University School of Medicine. Twenty-one male Fischer rats, weighing 190–220 g, were anesthetized by intraperitoneal injection of 10 mg/kg xylazine and 60 mg/kg ketamine. The head was clipped and prepared for aseptic surgery. Rats were immobilized in a small animal stereotactic headframe (Model 1350M, David Kopf Instruments, Tungisten, CA, USA). A lateral rostrotentorial surgical approach was made and an 8 mm×3 mm rectangular, parieto-occipital craniectomy defect was created in the right aspect of the skull using a high-speed Dremel drill. Custom, blunt-tipped IRE caliper electrodes were advanced into the cerebral cortex using stereotactic coordinates referenced to the location of the rostral electrode (bregma 4 mm posterior, 3.5 mm lateral, 1.5 mm dorsoventral). The caliper electrodes used were 0.45 mm in diameter, had 1 mm exposure, and were 4 mm in edge-to-edge separation distance.

The two-electrode configuration used generates a non-uniform electric field distribution that depends on the applied voltage and dielectric properties of the tissue. Therefore, we will refer to voltage-to-distance ratios in the manuscript in order to enable other researchers with the IRE pulse parameters used in the study. Animals underwent IRE treatment according to parameters in Table 1. To assess permeability of the BBB, Gd was administered to animals (n = 16) in each electric field group at varying times before or after delivering ninety 50-μs IRE pulses at a rate of one pulse per second (Table 1). Each animal received only a single contrast agent injection, with a separate animal being used to assess each time point and applied electric field. The control (sham) animals had the electrodes inserted into the brain but no pulses were delivered. One animal (n = 5) in each electric field group received Evan's Blue (50 mg/kg, IP) 5 minutes prior to IRE and was euthanized 30 minutes after pulse delivery without being subjected to magnetic resonance imaging (MRI) examination. Disruption of BBB, visible on histological sections in the animals was compared to the contrast-enhanced regions observed using MRI.

**Table 1:** Pulse parameters and Evan's Blue/Gd administration schedule used in IRE study.

Time (min)	0 V/cm	200 V/cm	400 V/cm	600 V/cm	800 V/cm	1000 V/cm
−5	† *		† *	† *	† *	† *
+5	*		*	*	*	*
+15		*		*		*
+30			*	*	*	

† =  Evan's Blue (n = 5); *  =  Gadolinium (n = 16) (Magnevist®). A separate animal was used to assess each time point and electric field (n = 21).

### Magnetic Resonance Imaging

A 7.0-T small animal MRI scanner (Bruker Biospec 70/30, Ettlingen, Germany) was used. Body temperature was maintained during scanning with thermostatically-controlled warm air. The heart rate, respiratory rate, and temperature were telemetrically monitored during scanning. A 38 mm inner diameter quadrature volume coil was used for RF signal transmission and reception (Litzcage, Doty Scientific, Columbia, SC). Sequence acquisition parameters were: T1-weighted (T1W) images were acquired using Rapid Acquisition with Relaxation Enhancement (RARE) pulse sequence with 8 echoes (TR = 1440 ms, TE = 7.5 ms, FOV = 4 cm, matrix = 256×256, slice thickness = 0.5 mm, NEX = 8), followed by the T2-weighted (T2W) images which were acquired using a RARE pulse sequence with 8 echoes (TR = 6575ms, TE = 60ms, FOV = 4 cm, matrix = 256×256, slice thickness = 0.5 mm, NEX = 8). T1W images were obtained following intraperitoneal administration of 0.1 mmol/kg of gadopentetate dimeglumine (Magnevist®: Bayer HealthCare Pharmaceuticals, Wayne NJ). The contrast was injected in reference to the delivery of the electric pulses according to the schedule in Table 1.

MR images were obtained 5–15 minutes after contrast injection which is consistent with where most of the enhancement from an IP injection of gadopentetate dimeglumine occurs as shown by Howles *et. al* [23]. Cross-sectional areas of contrast were contoured independently in a semi-automated manner on each slice of the T1W+Gd sequence, with volumes of contrast-enhancing tissue being calculated automatically with Mimics software 14.1 (Materialise, Leuven, BG). Because Gd is too large to cross the intact BBB in the cerebrum, any increase in contrast enhancement evident on T1W+Gd images, compared with levels of enhancement seen in the control animals, was taken as direct evidence of BBB disruption induced by IRE [5], [20], [21], [22]. T2W images were used to evaluate any edema surrounding the IRE ablated regions.

Contrast enhancement intensity was quantified using four reference tubes filled with known Gd concentrations (0, 0.09, 0.19, and 0.24 mg/ml) in saline and scanned with each rodent. A calibration curve was determined to allow for calculation of a normalized mean value of Gd concentration [24] within the IRE-induced volume of BBB disruption. Three mean intensity measurements within a 3.2 mm^2^ circle were averaged along the reference tubes in order to minimize the intensity variations within each scan. These independent measurements were performed on MRI slices that corresponded with the rostral and caudal electrode insertions and one slice in-between the electrodes. The mean intensity of each 3D reconstructed lesion was divided by the mean intensity of the 0.09 mg/ml reference tube in order to normalize the Gd concentration across the different treatments. The normalized intensities were then converted to Gd concentrations and are provided in the results section.

### Histopathology

An adult rat brain matrix slicer (Zivic Instruments, Pittsburg, PA) was used to obtain contiguous 3.0 mm coronal brain sections of formalin fixed brains. Brain sections were paraffin embedded, sectioned at 5 µm, and stained with hematoxylin-eosin (H&E). Each microscopic brain section was photographed at 150X magnification using a digital camera (Nikon DS-Fi1, Nikon, Japan). For each treatment and time, 3 separate independent hand drawn regions of interest (ROI) were traced around the boundaries of the IRE zone of ablation present in the brain, and the area of each ROI determined using the area function of image analysis software (NIS-Elements AR, Nikon, Japan).

**Figure 1:**
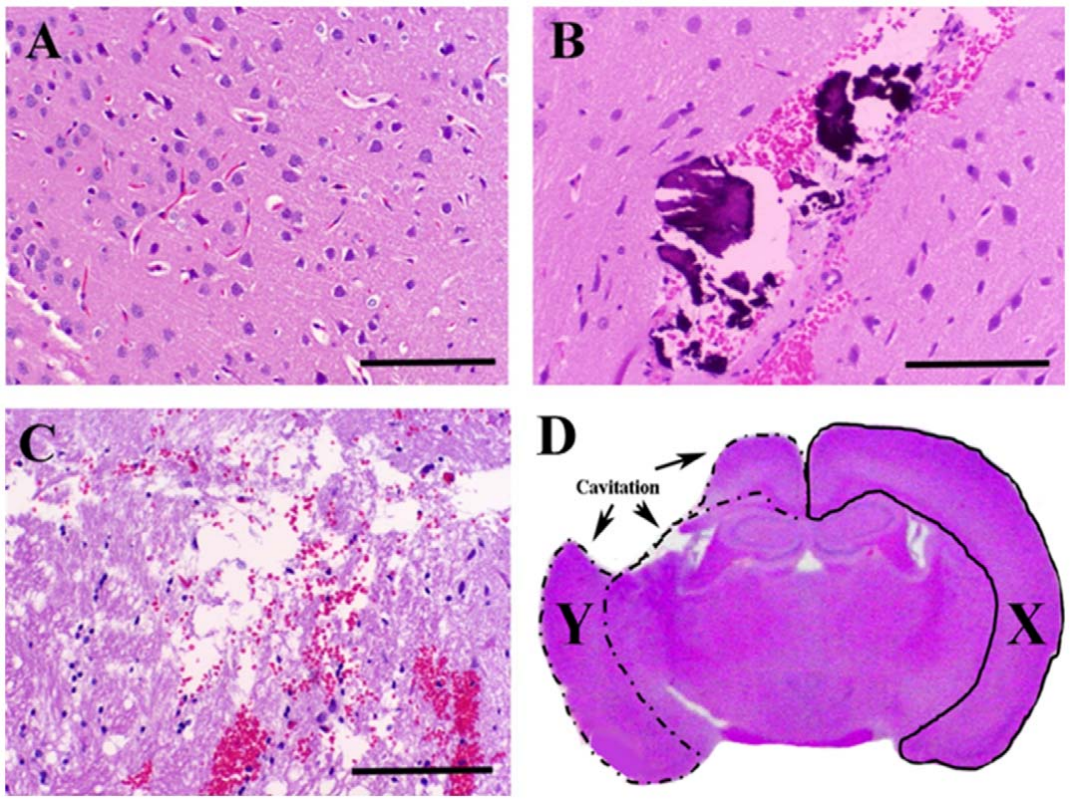
Histopathologic evaluation of IRE-induced effects determined with Hematoxylin and Eosin stain. Histopathologic sections of cerebral cortex from untreated control rat (**A**), sham treated rat with physical displacement of the neuropil in the trajectory of the electrode (**B**), and cortical ablation zone resulting from 800 V/cm IRE treatment (**C**). Histopathologic lesion area determination in presence of IRE induced cavitary cerebral defect (**D**). The IRE lesion area (mm^2^)  =  untreated cerebral area (X) – IRE lesioned cerebral area (Y). Bar  = 500 µm in panels A–C.

The ROI limits from which IRE zones of ablation were traced used the following anatomic boundaries: dorsal- dura mater, ventrolateral- inner limit of external capsule, ventromedial- inner limit of corpus callosum. Any intervening cerebrocortical tissue that was lesioned within these limits was included in the ROI. For sections in which IRE treatment resulted in a full thickness cerebrocortical defect or cavitation of tissue architecture, the lesion area was determined by subtracting the area of the cerebral hemisphere remaining intact on the IRE treated side of the brain (Fig. 1D–Y, dashed line) from the area of the contralateral (untreated; Fig. 1D–X, solid line) cerebral hemisphere. Cerebral hemispheric areas were determined from three separate hand-drawn ROI, using photomicrographs obtained at 50X magnification, as described above.

### Statistical Analysis

Statistical analysis on the effect of applied electric field and timing of Gd administration was conducted using JMP 9.0 (SAS, South Cary, NC) via Fit of Least Squares with α = 0.05. Linear regression analysis of the relationship between electric field and volume of ablation was also performed as it was found appropriate using previously published data [25].

## Results

On gross examination of the brain (Fig. 1), sham treatment resulted in two punctate, 1 mm cortical depressions corresponding to the points of electrode insertions. In histopathological sections, sham zones of ablation were characterized by physical displacement of the brain tissue along the electrode tracks and associated with microhemorrhage into the electrode tracks. Zones of ablation in sham-treated rats were limited to the immediate proximity of the electrode insertions, with the adjacent neuropil retaining normal cortical architecture and morphology (Fig. 1A). At voltage-to-distance ratios of 200 V/cm and 400 V/cm (Fig. 1B), observed gross and histopathological zones of ablation were morphologically indistinguishable from those of sham-treated rats. At voltage-to-distance ratios greater than 600 V/cm, IRE treatment resulted in distinct areas of parenchymal ablation (Fig. 1C). Grossly, ablated regions were malacic. Microscopically, IRE zones of ablation were characterized by an eosinophilic, vacuolated amorphous debris and multifocal areas of intraparenchymal hemorrhage, consistent with coagulative necrosis. Variably-sized regions of intraparenchymal hemorrhage were noted; these were most pronounced immediately adjacent to and within electrode insertion tracks similar to previous results in canine brain [16]. Remnant neurons within ablated regions were shrunken, had hypereosinophilic cytoplasm and showed nuclear pyknosis and/or karyolysis. Free glial nuclei in various states of degeneration were scattered throughout ablation zones.

**Figure 2:**
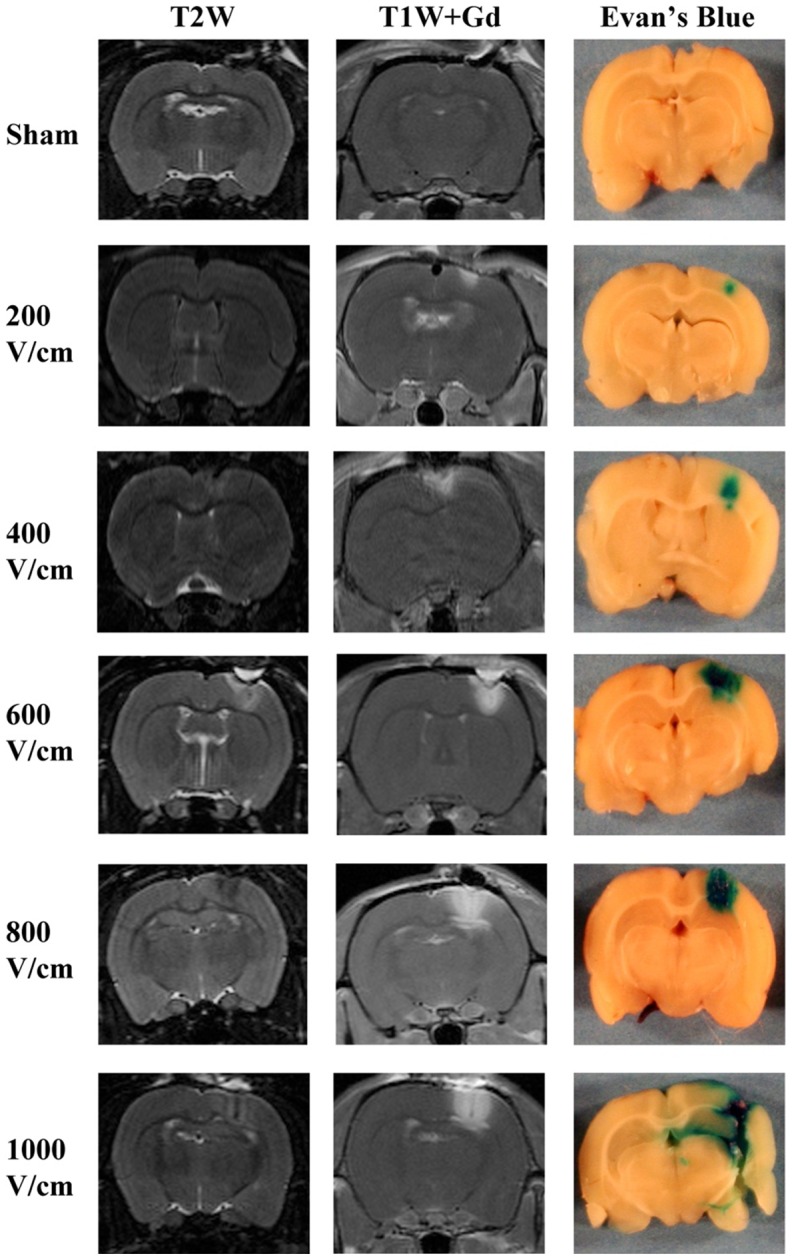
Morphologic characteristics of IRE-induced BBB disruption on 7.0-T MRI and Evan's Blue brain sections. The zones of ablation were achieved with ninety 50-μs pulses at a rate of one pulse per second. The Gadolinium (Gd) and Evan's Blue dyes were administered IP 5 minutes before the delivery of the pulses. The positive correlation between the applied voltage-to-distance ratios and the extent of BBB disruption induced by IRE is indicated by the uniformly contrast-enhancing zones of ablation on the T1W+Gd MR images and corresponding Evan's Blue brain slices. IRE-induced zones of ablation are sharply demarcated from the surrounding brain parenchyma. Linear hypointensities in the center of the zones of ablation, corresponding to the electrode insertions, are evident in the MR images from the 600, 800, and 1000 V/cm treatments.

All treatments resulted in zones of ablation visible on MRI (Fig. 2 and Movie S1). In sham-treated rats in which the electrodes were inserted into the brain but no pulses applied, zones of ablation were limited to physical displacement of the brain parenchyma, which appeared as hypointense electrode tracks on T1W+Gd and T2W sequences (Fig. 2). No contrast enhancement or intraparenchymal uptake of Evan's blue in the adjacent brain was observed in sham-operated rats (Fig. 2). At all voltage-to-distance ratios examined, IRE treatment induced heterogeneous T2W zones of ablation characterized by a hypointense central lesion with perilesional T2W hyperintensity (Fig. 2) and markedly and uniformly contrast-enhancing zones of ablation that were sharply delineated from the adjacent brain tissue (Fig. 2).

**Figure 3:**
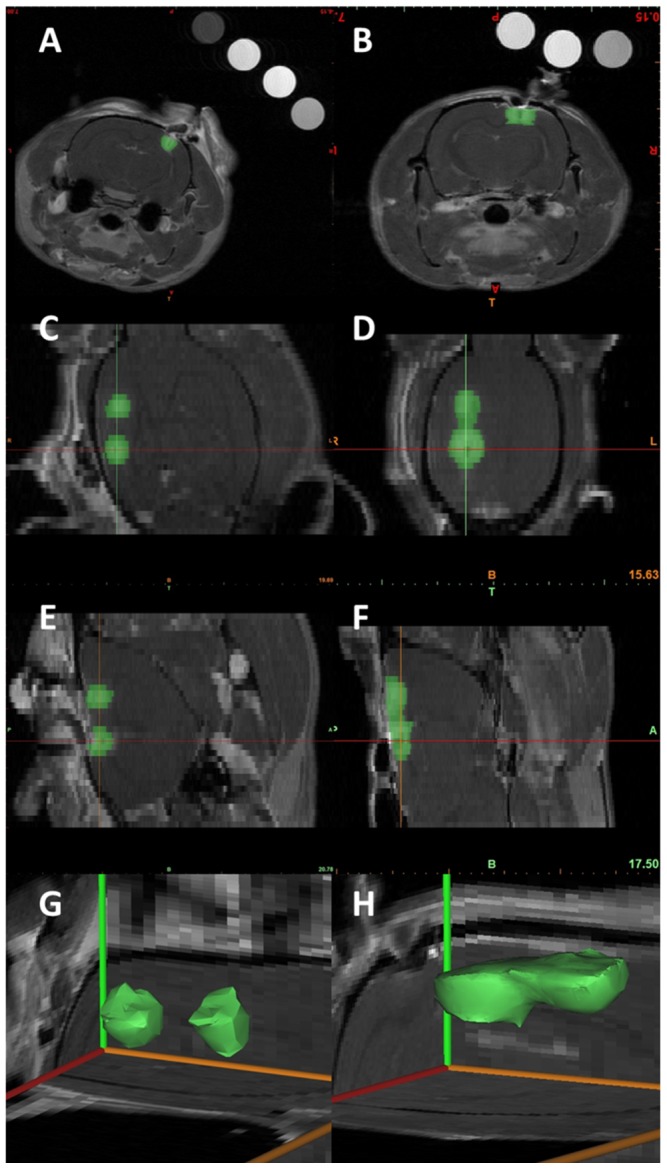
Qualitative representations of IRE-induced BBB disruption using 7.0-T MRI. 2D IRE lesion tracing on the coronal (**A, B**), dorsal (**C, D**), and sagittal (**E, F**) planes with the corresponding non-contiguous (**G**) and contiguous (**H**) 3D reconstruction zones of ablation representative of 400 V/cm and 1000 V/cm IRE treatments, respectively. These reconstructions illustrate the shapes of the IRE zones of ablation, which are consistent with the electric field distributions that would be generated with the electrode configuration and pulse parameters used in this study. By optimizing treatment protocols and electrode configurations, it is possible to disrupt the BBB to target different size and shapes of tissue.

On MRI scans (Fig. 3), treatment at 200 V/cm and 400 V/cm induced two non-contiguous ovoid to spherical IRE zones of ablation centered around the electrodes tips (Fig. 3A, 3C, 3E), with the largest cross-sectional area in the coronal plane along the electrode tract. 3D reconstructions demonstrated two separated spherical regions surrounding the 1-mm electrodes (Fig. 3F). IRE treatment at 600 V/cm, 800 V/cm, and 1000 V/cm resulted in a “peanut shape” lesion that was contiguous between the two electrodes (Fig. 3B, 3D, 3F), with similar characteristics to the 3D reconstruction in Fig. 3H. The different reconstructed geometries for each applied voltage confirm the electric-field dependent effect of IRE. In addition, these results suggest that the threshold for IRE-induced BBB disruption is between 400 V/cm and 600 V/cm which is consistent with previous studies in brain [17].

**Figure 4:**
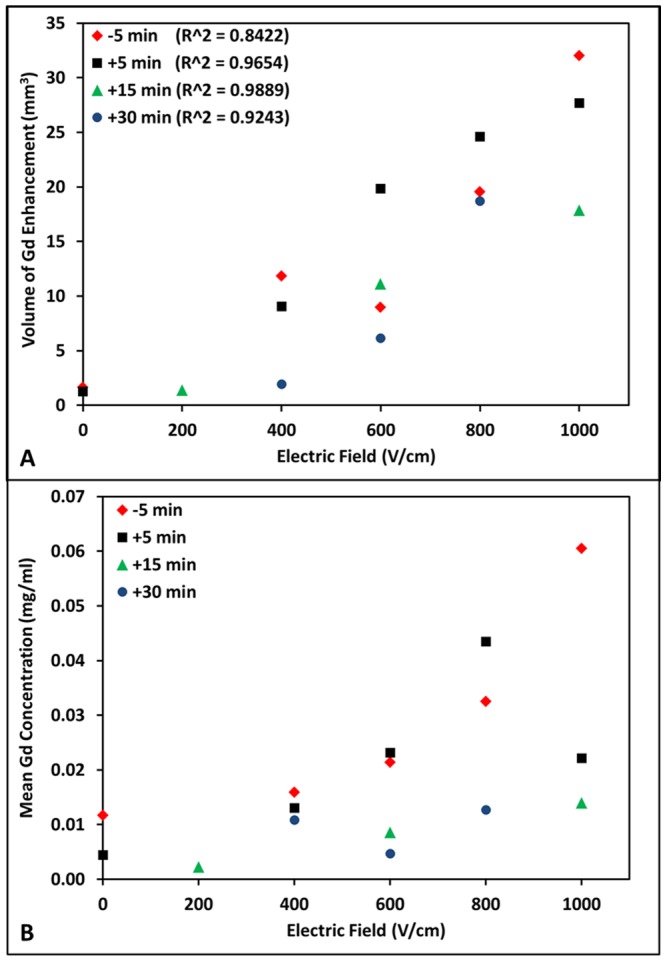
Quantification of IRE-induced BBB disruption from the 3D MRI reconstructions. Volumes (**A**) and mean concentrations (**B**) of Gd enhancement are provided as a function of the applied voltage-to-distance ratio and timing of Gd administration. Although N is low, the finding of increasing volume of affected tissue with increasing voltage applied (for the electrode configuration and pulse parameters used) suggests that volume is directly related to voltage. Similarly, the finding of a trend toward decreased volumes with increasing delays after Gd administration suggests a possible transient quality to the permeabilization surrounding the regions of ablation. The linear fit used to correlate the electric field and zone of ablation was found appropriate using previously published data [25]. The mean concentrations of Gd within the reconstructed IRE-induced regions of BBB disruption are also positively correlated with the applied electric field. This is a critical observation since it provides evidence that with increasing electric field strengths even more electroporation is achieved and transport of Gd or other exogenous agents is enhanced.

With histopathologic (Fig. 1), Evan's Blue (Fig. 2), and MRI (Fig. 3) examinations, the extent of BBB disruption was positively correlated with the applied voltage-to-distance ratio. Objective measurements confirming this are provided in Fig. 4, in which the volumes of gadolinium (Gd) enhancement and mean concentrations are plotted as a function of the applied voltage-to-distance ratio and timing of Gd administration. Within each time points in which Gd was administered, linear correlations were determined between electric fields and volumes of ablation (−5 min: R^2^ = 0.8422, +5 min: R^2^ = 0.9654, +15 min: R^2^ = 0.9889, +30 min: R^2^ = 0.9243). There was a significant positive correlation of Gd volume (p<0.0001) and mean concentration (p = 0.0077) with applied electric field. The negative correlation of Gd volume (p = 0.0151) and concentration (p = 0.0056) with time was also statistically significant, confirming the transient permeabilization surrounding the regions of ablation. Exposing the brain tissue to increasing applied electric fields resulted in larger volumes of Gd enhancement.

Cross sectional areas of Gd enhancement around the rostral electrode were also calculated, to compare the results from the T1W+Gd MRI with the corresponding cross-sectional areas seen in the histopathology and gross pathology specimens along the coronal plane. Table 2 shows cross-sectional areas of Gd enhancement from the MRI, cross-sectional areas of IRE cell death derived from H&E images, and cross-sectional areas of permeabilization from the Evan's Blue. The cross-sectional areas of Gd enhancement (p<0.0001) and cell death (p<0.0001) surrounding the rostral electrode were evaluated via Standard Least Square Fit and showed a significant positive correlation with the applied electric field. No significant correlation was found between the cross-sectional areas of MRI enhancement and timing of Gd administration. The results indicate that the cross-sectional areas of Gd enhancement and Evan's Blue are predominantly greater than the cell death cross-sectional areas from the H&E stained sections, confirming the existence of the penumbra of transient BBB disruption.

**Table 2:** Resulting IRE-induced BBB disruption mean concentrations, volumes, and cross-sectional areas calculated using the Gd enhancement in MRI, H&E, and Evan's Blue.

Time (min)	E-Field (V/cm)	Mean Gd Conc. (mg/ml)	Gd (MRI) (mm^3^)	Gd (MRI) (mm^2^)	H&E (mm^2^)	Evan's Blue (mm^2^)
−5	0	0.012	1.62	0.00	1.31	
−5	400	0.016	11.82	1.56	1.52	3.22
−5	600	0.021	8.97	4.22	3.14	3.98
−5	800	0.033	19.55	5.15	4.83	4.29
−5	1000	0.060	32.00	6.67	4.51	4.97
+5	0	0.004	1.26	0.46	1.14	
+5	400	0.013	9.07	3.66	2.08	
+5	600	0.023	19.83	4.05	3.82	
+5	800	0.043	24.61	5.25	3.04	
+5	1000	0.022	27.69	4.91	5.84	
+15	200	0.002	1.39	1.22	1.39	
+15	600	0.009	11.13	4.79	3.39	
+15	1000	0.014	17.85	4.79	5.46	
+30	400	0.011	1.93	1.17	1.09	
+30	600	0.005	6.16	2.25	1.68	
+30	800	0.013	18.71	6.25	4.63	

**Note: The cross-sectional areas were determined from the regions intersecting the rostral electrode tip.**

## Discussion

Using rodents as predictive models, we present data on the duration and extent of acute BBB disruption surrounding an IRE-induced zone of ablation. We hypothesize that there is a minimal electric field at which BBB disruption occurs surrounding an IRE-induced zone of ablation and that this transient response can be measured using Gd uptake as a surrogate marker for BBB disruption. To test this hypothesis, we performed IRE at different electric fields and varied the timing of Gd administration to estimate the duration of any reversible effects on BBB disruption. The results show the minimal pulse parameters necessary for effective BBB disruption and provide an estimate of the duration and extent of reversible effects on BBB disruption, supporting our hypothesis of the creation of a non-destructive penumbra of BBB disruption adjacent to regions of IRE-induced cell death.

We observed IRE-induced BBB disruption over the entire range of electric field strengths evaluated. These results provide preliminary guidelines for electric field thresholds for IRE in the normal rodent brain. Histopathological examinations were consistent with previous pathological descriptions of IRE-induced cerebrocortical ablations [16], [17], at applied voltage-to-distance ratios greater than 600 V/cm, while the morphology of brain tissue treated at voltage-to-distance ratios smaller than 400 V/cm was identical to sham-treated rodents. This indicates electroporation is predominantly or exclusively reversible at electric field strengths <400 V/cm in normal brain, using the pulse parameters applied in this study. The extent of reversible BBB disruption induced by IRE is underestimated using methods based on H&E stained sections, when compared to paramagnetic contrast agents or vital dye surrogates of BBB disruption. Co-labeling methods, that simultaneously utilize both imaging contrast agents and vital dyes, would be recommended in future studies to define the reversible electroporation domain in brain tissue [26].

In BBB permeability modeling, the parenchymal uptake of low molecular weight, paramagnetic positive contrast imaging agents, such as Gd, is representative of solute and ion uptake during BBB disruption, while uptake of higher molecular weight, protein-bound vital dyes, such as Evan's Blue, indicate increased BBB permeability to protein. The observation of brain uptake of both Evan's Blue and Gd in all treatment groups indicates that IRE results in BBB permeability to solutes, ions, and protein, but the discrepancies observed in the lesion sizes as determined with Gd and Evan's Blue qualitatively suggest that BBB permeability induced by IRE is non-uniform. BBB permeability is likely a transient and dynamic process given the electroporation of tissue and disruption of microvascular blood flow that have been shown to occur during delivery of electric pulses [16], [17], [25], [27].

We recognize limitations of our study, including small sample size and the lack of neuromuscular blockade, which is traditionally used in IRE therapies. The goal of the pilot study was to preserve animals and cost and still be able to get a sense of the timing and electric field strength needed for BBB disruption. Minimal motion in the head of subjects was noted. The influence of the vertical motion on the volumes of enhancement was minimized by restricting the calculation of the IRE-mediated BBB disruption to the brain regions described in the Methods section. It is possible that electrodes may have not been in identical locations for each rat, which could have resulted in ablation regions being outside targeted anatomical boundaries. We evaluated the effect only in normal rodent brains; it is possible that brain tumors respond differently to differing voltages of IRE. In spite of these limitations, we were able to demonstrate significant correlations between the applied electric field and the timing and extent of Gd enhancement. Our results also suggest there is a persistent effect on BBB disruption at +15 and +30 minutes post-treatment. This time frame may prove to be advantageous in that it may be feasible to use more advanced, perfusion-based MRI techniques in future studies with IRE in the brain [28].

This preliminary study was performed to demonstrate IRE-induced BBB disruption and cell death using high-resolution MRI and histopathological evaluation. We investigated the IRE pulse parameters that we are currently employing in a pre-clinical trial of canine patients (Ninety 50-μs pulses delivered at 1 Hz) with spontaneous brain cancer [18]. Future studies are needed to compare IRE to recently developed non-invasive techniques to disrupt the BBB including focused and unfocused ultrasound [5], [23]. IRE is a non-thermal ablation technique that kills tissue in a focal manner depicted by MR imaging and transiently disrupts the BBB adjacent to the ablated area in a voltage-dependent manner. Future work will also investigate the effects of pulse duration and pulse number on blood-brain-barrier disruption and will determine electric field thresholds for reversible and irreversible electroporation. Incorporating the data presented here in computational models of the rodent brain could be used to develop intracranial electroporation-based treatment paradigms.

## Supporting Information

Movie S1 **IRE-induced BBB disruption as seen in the T1W+Gd MRI.** The lesion was achieved with ninety 50-μs pulses at 1000 V/cm with Gd administration 5 min pre-IRE at a repetition rate of one pulse per second.
(ZIP)
